# Transient elevation of squamous cell carcinoma antigen levels with influenza virus infection

**DOI:** 10.1002/rcr2.362

**Published:** 2018-09-14

**Authors:** Atsushi Sano

**Affiliations:** ^1^ Department of Thoracic Surgery Chigasaki Municipal Hospital Japan

**Keywords:** Influenza virus, squamous cell carcinoma, tumour marker

## Abstract

We report a case of squamous cell carcinoma antigen (SCCA) elevation due to influenza B infection. A 78‐year‐old male had undergone right middle lobectomy and lymphadenectomy for lung squamous cell carcinoma two years and four months previously. His SCCA level ranged from 0.8 ng/mL to 1.9 ng/mL after the surgery. He underwent blood testing, including SCCA, as part of a regular check‐up three days after the diagnosis of the influenza B infection. His SCCA level was 17.1 ng/mL; no recurrences were found on computed tomography. One month later, his SCCA level had decreased to 1.6 ng/mL. We should keep in mind that influenza infection may cause transient elevations in SCCA levels.

## Introduction

Squamous cell carcinoma antigen (SCCA) is a glycoprotein that is used as a tumour marker for squamous cell carcinoma of the neck, oesophagus, lung, and cervix uteri 1. SCCA levels also become elevated due to atopic dermatitis, bronchial asthma, and other conditions 2, 3, 4, 5, 6. Here, we report a case of SCCA elevation due to influenza B infection in a patient with a history of surgery for lung cancer.

## Case Report

A 78‐year‐old male with a history of early gastric cancer and meningioma of the brain underwent right middle lobectomy and lymphadenectomy for squamous cell lung carcinoma two years and four months ago. After the surgery, he received adjuvant chemotherapy with tegafur‐uracil fortwo2 years. He underwent regular check‐ups, including computed tomography and SCCA. He was doing well without recurrence of cancer. His SCCA levels ranged from 0.8 ng/mL to 1.9 ng/mL.

**Figure 1:**
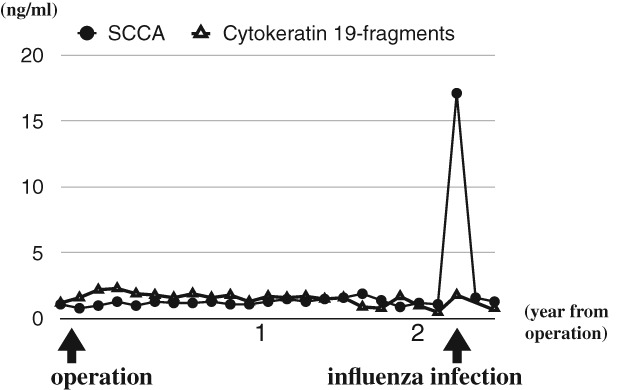
Levels of squamous cell carcinoma antigen (SCCA) (normal range, 0–1.5 ng/mL) and cytokeratin 19 fragments (normal range, 0–3.5 ng/mL) over time. After the diagnosis of lung cancer, SCCA levels were low. Three days after the diagnosis of influenza, SCCA levels increased. three weeks later, SCCA levels decreased. The levels of cytokeratin 19 fragments were low throughout this period.

He underwent blood testing including SCCA as part of a regular check‐up six days after getting a fever. He was diagnosed with influenza B infection three days before the blood test. He did not take any anti‐influenza drugs. His fever resolved by day 5. His SCCA level was 17.1 ng/mL. Computed tomography of the chest and abdomen revealed neither recurrence of cancer nor new lesions. We suspected that the SCCA level became elevated due to the influenza infection. One month later, his SCCA level decreased to 1.6 ng/mL (Fig. 1). Levels of cytokeratin 19 fragments remained low throughout this period.

## Discussion

SCCA is a representative tumour marker of squamous cell carcinoma that is often used for the diagnosis and follow up of squamous cell carcinoma originating from various organs. On the other hand, SCCA is a non‐specific test. SCCA levels also become elevated due to atopic dermatitis, bronchial asthma, pulmonary tuberculosis, and other conditions 2, 3, 4, 5, 6. Mitsuishi et al. reported that SCCA is induced in skin with atopic dermatitis through the direct action of interleukin (IL)‐4 and/or IL‐13 on keratinocytes 2. Nishi et al. reported that IL‐13 might contribute to elevations in SCCA in patients with bronchial asthma 3. Therefore, immune reactions might contribute to elevations in SCCA.

Only one case of SCCA elevation due to influenza infection has been reported so far. Fujioka et al. reported a case of elevated SCCA in a patient with influenza virus infection 7. Their patient presented with generalized fatigue and was then diagnosed with influenza infection. His serum SCCA level was elevated at 14.4 ng/mL. No malignant tumours were found, and his serum SCCA level decreased to less than 1.0 ng/mL after 3 weeks.

Similarly, our patient had a transient SCCA elevation to a value approximately 10 times the normal value during influenza virus infection; the SCCA level soon decreased. In our case, it was confirmed that the SCCA level before influenza virus infection was low. Therefore, we speculate that the transient elevation in SCCA levels was caused by influenza virus infection.

As there have been only a few reports, the mechanisms underlying SCCA level elevation remain unknown. Elevations in SCCA might be due to the direct action of the influenza virus or immune reactions related to IL‐4 or IL‐13. It is unknown whether influenza infection always causes elevations in SCCA. Information from more cases is needed to understand this potential association.

In conclusion, we should keep in mind that influenza infection might cause transient elevations in SCCA levels.

## Disclosure Statement

Appropriate written informed consent was obtained for publication of this case report and accompanying images.
